# The Occurrence of Flavonoids and Related Compounds in Flower Sections of *Papaver nudicaule*

**DOI:** 10.3390/plants5020028

**Published:** 2016-06-22

**Authors:** Bettina Dudek, Anne-Christin Warskulat, Bernd Schneider

**Affiliations:** Max Planck Institute for Chemical Ecology, Hans-Knöll-Straße 8, 07745 Jena, Germany; bdudek@ice.mpg.de (B.D.); awarskulat@ice.mpg.de (A.-C.W.)

**Keywords:** flower pigmentation, flavonoids, kaempferol, pelargonidin, nudicaulins, *Papaver nudicaule*

## Abstract

Flavonoids play an important role in the pigmentation of flowers; in addition, they protect petals and other flower parts from UV irradiation and oxidative stress. Nudicaulins, flavonoid-derived indole alkaloids, along with pelargonidin, kaempferol, and gossypetin glycosides, are responsible for the color of white, red, orange, and yellow petals of different *Papaver nudicaule* cultivars. The color of the petals is essential to attract pollinators. We investigated the occurrence of flavonoids in basal and apical petal areas, stamens, and capsules of four differently colored *P. nudicaule* cultivars by means of chromatographic and spectroscopic methods. The results reveal the specific occurrence of gossypetin glycosides in the basal spot of all cultivars and demonstrate that kaempferol glycosides are the major secondary metabolites in the capsules. Unlike previous reports, the yellow-colored stamens of all four *P. nudicaule* cultivars are shown to contain not nudicaulins but carotenoids. In addition, the presence of nudicaulins, pelargonidin, and kaempferol glycosides in the apical petal area was confirmed. The flavonoids and related compounds in the investigated flower parts and cultivars of *P. nudicaule* are profiled, and their potential ecological role is discussed.

## 1. Introduction

Ubiquitous in angiosperms, flavonoids are extremely diverse in their chemical structure, color, and biological function. Flavonols, flavones, flavanones, flavanols, and anthocyanidins are just a few examples of the wide range of subclasses [1]. Anthocyanidins, in particular, and their corresponding glycosides (anthocyanins) are flower and fruit pigments, which enhance pollination and seed dispersal [2]. These red to blue pigments are often accompanied by pale yellow or colorless flavonols, which serve as co-pigments and may play a role in UV protection, disease resistance, or hormone signaling [3]. In flowers, the occurrence and distribution of flavonoids is likely connected to their specific function.

In 1931, the first flower pigments of *Papaver nudicaule*, a poppy species originating from Siberia [4] but commonly known as the Iceland poppy, were identified as pelargonidin glycosides in red and orange petals [5,6]. The specific substitution patterns of these anthocyanins were investigated later on by means of mass spectrometry (MS) and nuclear magnetic resonance (NMR) spectroscopy [7,8].

**Figure 1:**
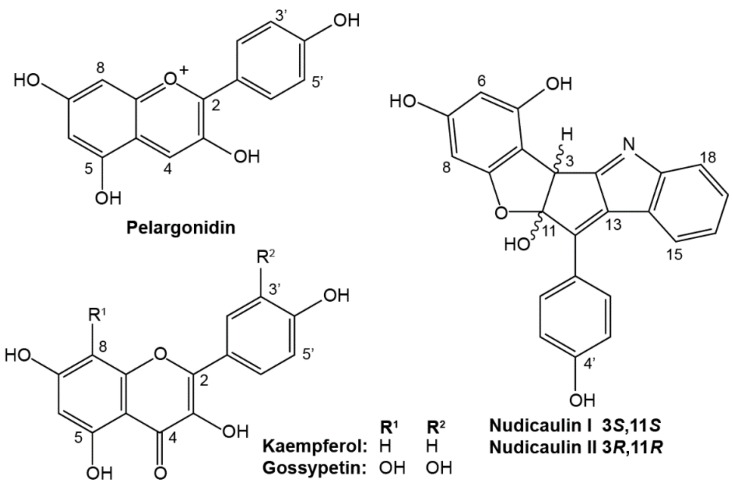
Aglycone structures of flavonoids and nudicaulins from *P. nudicaule* flowers.

Yellow and orange flowers of *P. nudicaule* contain the yellow nudicaulins, an unusual group of indole alkaloids with three glucose substituents (Figure 1), that are derived from the indole and flavonoid biosynthetic pathways [8,9]. According to Schliemann et al. (2006), in yellow petals, nudicaulins are accompanied by gossypetin 7-*O*-glucoside (gossypitrin) and seven kaempferol glycosides whose substitution patterns correspond to those of the indole alkaloids [10]. In the absence of other pigments, the colorless kaempferol glycosides and the pale yellow gossypitrin contribute to the white or ivory appearance of petals [11]. Additionally, the presence of kaempferol in pollen was linked to the production of functional pollen tubes and to a successful germination process in maize and petunia, but its absence does not automatically imply sterility [12].

In 1962, it was shown that yellow compounds are present in both the petals and stamens of wild-type and cultivars of *P. nudicaule*; pelargonidin glycosides were limited to the petals of garden varieties [13]. Furthermore, a yellow compound that was named nudicaulin was reported in the filament of stamens in various *Papaver* species, but there was no accompanying detailed chemical analysis [14].

**Figure 2:**
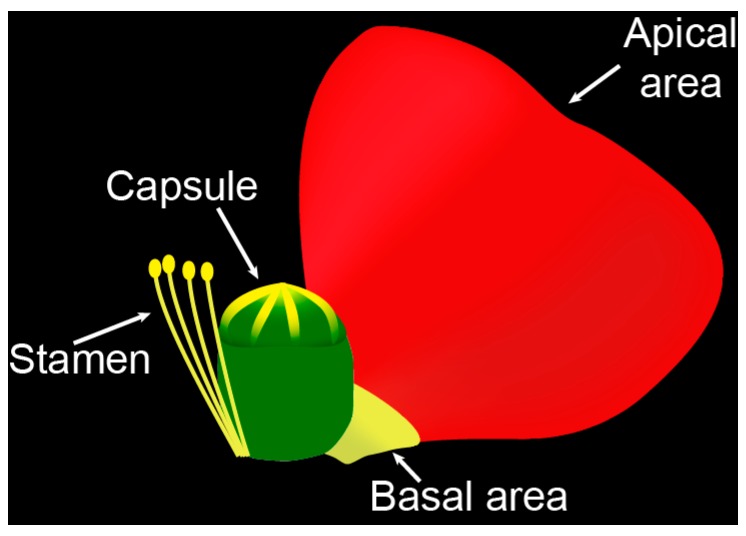
Scheme of a *P. nudicaule* flower and the four investigated flower parts.

In the present study, we report on the occurrence of flavonoids and the biosynthetically related nudicaulins in two petal areas, capsules, and stamens (Figure 2) of white, yellow, orange, and red flowers of *P. nudicaule* cultivars. The potential biological and ecological function of the constituents of *P. nudicaule* is discussed in the context of current knowledge and hypothetical considerations.

## 2. Results

### 2.1. Apical and Basal Petal Areas

**Figure 3:**
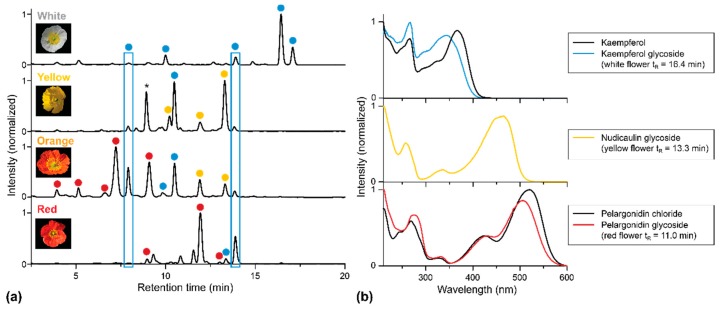
HPLC-PDA analysis of extracts of apical petal parts of four *P. nudicaule* cultivars. (**a**) Chromatograms recorded at 254 nm. Peaks representing the same aglycone (previously identified by LC-MS and NMR [7,10,15] and here classified by the corresponding UV/Vis absorption spectra) are marked with the same color: ● Kaempferol glycoside, ● nudicaulin, ● pelargonidin glycoside. The peak marked with an asterisk (yellow flower, t_R_ = 8 min) shows no flavonoid absorption spectrum and may be a degradation product. (**b**) UV/Vis absorption spectra of representative glycosides and authentic aglycones of kaempferol and pelargonidin. Deviations between UV/Vis absorption spectra of the references (kaempferol, pelargonidin chloride), and the glycosides are an effect of the substitution. Nudicaulin aglycone is not available due to instability. The obtained nudicaulin UV/Vis absorption spectrum matches the one reported by Tatsis et al. [15].

High-performance liquid chromatography–photodiode array detection (HPLC-PDA) analysis of fresh extracts obtained from the apical petal areas of the white, yellow, orange, and red cultivars confirmed the presence of nudicaulins, kaempferol, and pelargonidin glycosides. In previous studies, the aglycone structures and the substitution patterns of the apical pigments were already elucidated by LC-MS (Figure S1) and NMR [7,10,15]. Taking this knowledge into account and considering the analytical value of characteristic UV/Vis absorption spectra, we suppose that the major flavonoid substance classes are those shown in Figure 3. These UV/Vis absorption spectra were also used to characterize the compounds in extracts of the other flower parts. Based on these data, kaempferol glycosides are assumed to be the only flavonols occurring in the apical petal area of all *P.nudicaule* cultivars. Two of these glycosides are present in all flower samples (indicated by blue boxes in Figure 3). Likewise, pelargonidin glycosides are assumed to be the only group of anthocyanidins in the flowers of this plant.

White flowers possess the most kaempferol glycosides but lack pelargonidin glycosides and nudicaulins. The two major kaempferols elute after a retention time (t_R_) of 16 min, indicating a lower polarity and a reduced degree of glycosylation compared with kaempferol glycosides occurring in petals of other cultivars.

Furthermore, the nudicaulins are present exclusively in yellow and orange flowers, where they have identical t_R_ and, consequently, identical substitution patterns. This confirms previous studies [8]. In contrast, pelargonidin glycosides occur solely in orange and red petals. Due to varying substituents, the pelargonidin glycosides found in red flowers are less polar than those found in orange ones, which is consistent with reported data [15].

**Figure 4:**
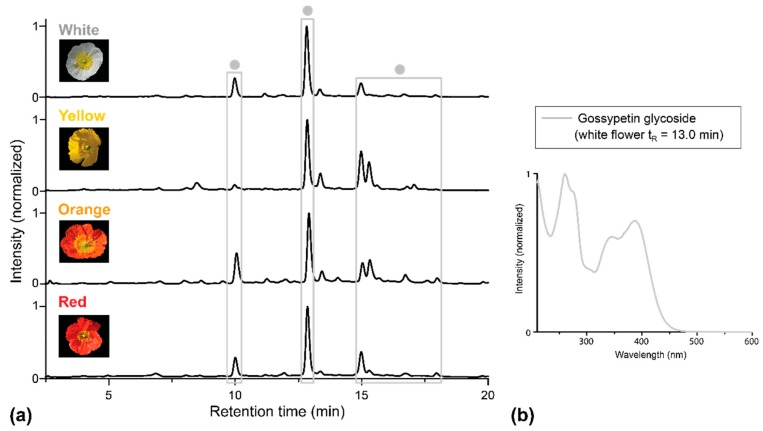
HPLC-PDA analysis of extracts of basal petal parts of four *P. nudicaule* cultivars. (**a**) Chromatograms recorded at 351 nm. Gossypetin glycosides (identified by UV/Vis absorption spectra) are marked with dots ●. (**b**) UV/Vis absorption spectrum of one representative gossypetin glycoside. The spectrum matches the one reported by Suzuki et al. [16].

Although the basal petal part appears yellowish, the presence of nudicaulins in this tissue was not confirmed. The only flavonoids present in this area, gossypetin glycosides, are absent in the apical part of the flowers. Overall, the HPLC-PDA profiles of the basal petal areas of all four *P. nudicaule* cultivars are very similar (Figure 4).

### 2.2. Stamens and Capsules

**Figure 5:**
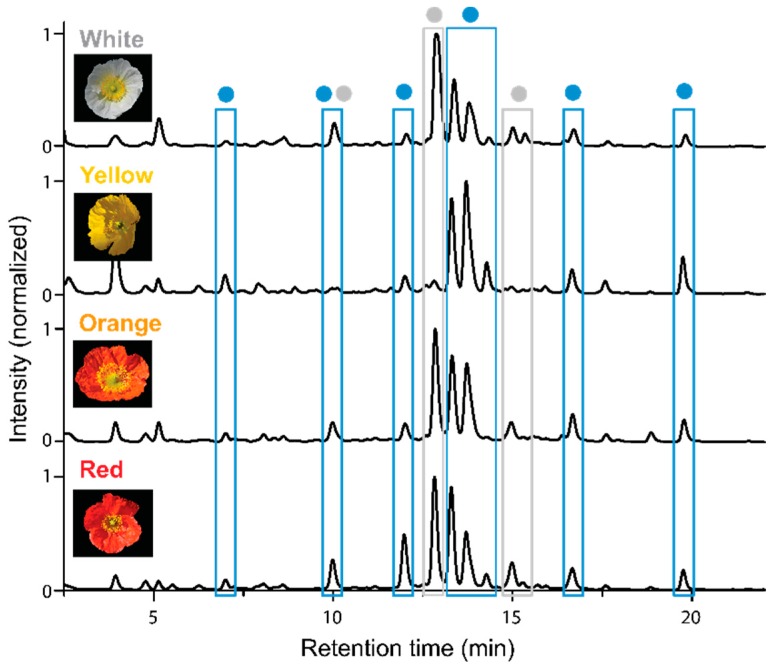
HPLC-PDA chromatograms of stamen extracts of four *P. nudicaule* cultivars recorded at 254 nm. Peaks with the same aglycone (identified by UV/Vis absorption spectra) are marked with the same color: ● Kaempferol glycoside, ● gossypetin glycoside.

The stamen extracts contain a huge diversity of flavonoids or, more precisely, flavonols (Figure 5). In this flower section, kaempferol and gossypetin glycosides occur side by side, and all four *P. nudicaule* cultivars possess the same substances. Pelargonidin glycosides and nudicaulins are missing in the stamens.

Extracts of the capsules of all four cultivars contain only traces of kaempferol glycosides, indicating that flavonoids likely do not serve a function in this area or are only present at an earlier stage of capsule development. Moreover, because the yellow pigments of stamens and the upper area of the capsule could not be extracted from the tissue by a water-methanol mixture (Method 1; see Section 4.2), it was unlikely that nudicaulins are responsible for the yellow color of these tissues.

**Figure 6:**
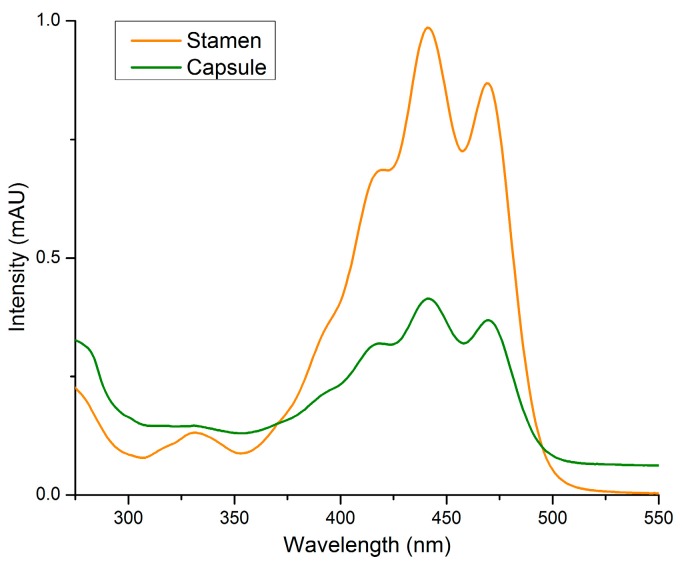
UV/Vis absorption spectra of pigments obtained by extraction with hexane from stamens and capsules (Method 2). The spectra of extracts of yellow flower parts are shown as representative examples for all cultivars.

When hexane was used as an extractant (Method 2; see Section 4.3), it was possible to retrieve the yellow color from stamens and the upper capsule. As expected, the UV/Vis absorption spectra did not match with those of the flavonoids and nudicaulins obtained from the other flower parts. In contrast, the UV/Vis spectra corresponded to the known absorption characteristics of carotenoids (Figure 6) [17]. We conclude that carotenoids serve as yellow pigments in the stamens and upper capsules of all four *P. nudicaule* cultivars.

## 3. Discussion

This study investigated the occurrence of UV/Vis absorbing pigments in the apical and basal petal areas, capsules, and stamens of *P. nudicaule* flowers. Glycosides of two flavonols (i.e., kaempferol, gossypetin), one anthocyanidin (i.e., pelargonidin), and flavonoid-derived indole alkaloids (nudicaulins), as well as carotenoids were detected. Their distribution in different flower parts of the examined white, yellow, orange, and red cultivars is summarized in Table 1.

Pelargonidin glycosides and nudicaulins are present in the apical parts of red, orange, and yellow *P. nudicaule* flowers. This is in agreement with results from previous studies [7,8,10].

Since pelargonidin glycosides represent biosynthetic precursors of the nudicaulins [9], the fact that orange and red cultivars contain different pelargonidin glycosides raises questions: Does the substitution pattern affect the conversion to nudicaulins or is the formation of nudicaulins limited by the availability of indole, the second ultimate biosynthetic precursor?

The identified kaempferol glycosides are the main flavonoids in the apical area of white petals and may function as UV pigments, as reported for other plants [11]. Additionally, kaempferol glycosides could serve as UV screens in the petal tissue, although the lack of a second hydroxyl group in ring B renders them less optimal for such a function [18]. Since in all four cultivars these compounds are produced in the developing petals before the buds open for flowering, it is more likely that the kaempferol glycosides play a role in growth processes than in the protection of the plants against UV light. In addition, in yellow-, orange- and red-colored petals, kaempferol glycosides might act as co-pigments [19].

Pigments coloring the apical area serve an important ecological function by attracting pollinators such as bees and bumblebees. Differently appearing petals might attract different pollinators and therefore improve reproductive success [2]. Furthermore, because the flowers of the *Papaver* plants are open during both day and night, a broad pollinator audience with varying color preferences may be attracted. Consequently, the various flower constituents in *P. nudicaule* cultivars may affect different processes, each of which contributes to the survival of the species.

The present study describes for the first time the occurrence of several gossypetin glycosides exclusively in the basal area of the petals of all four cultivars. Until now, only gossypitrin, the gossypetin 7-*O*-glucoside, had been reported in yellow petals [10]. The fact that we detected more gossypetin glycosides than Schliemann et al. (2006) is considered to be a consequence of our modified experimental protocol, which includes separate extractions of basal and apical petal areas [10]. Gossypitrin is responsible for the yellow color of *Chrysanthemum segetum* flowers and, along with herbacitrin (missing OH group in position 3′), of *P. radicatum* [20,21]. It is unlikely, however, that gossypetin glycosides serve primarily as pigments in the basal area of *P. nudicaule*, because the basal flower part is visually covered by stamens and the capsule. Similarly, the functions as a UV protector (for which gossypetins would have the catechol moiety as a favorable structural precondition) or as a visual nectar guide for insects, as described for example in *Rudbeckia hirta* [22], are not likely.

However, it is noticeable that gossypetin glycosides also occur, together with kaempferol glycosides, in the stamens of all four *P. nudicaule* cultivars. It may be that in *P. nudicaule* the found flavonols play a role in pollen germination or pollen tube development, as it was previously reported in maize and petunia [23].

Furthermore, contrary to preceding reports [14], we show that carotenoids, not nudicaulins, are coloring the yellow stamen and the upper part of the capsule. Carotenoids are widespread pollen and stamen pigments and are also known from some poppy species, for example, *Eschscholzia californica* Cham. [24]. The yellow pigments of stamens of various *Papaver* species—tentatively reported by Tétényi to be nudicaulins—may have been confused with these indole alkaloids because of their similar color. As of now, nudicaulins are known only from petals of *P. nudicaule*, *Papaver alpinum*, and *Meconopsis cambrica* [8].

Our results show a defined distribution of weakly colored kaempferol and gossypetin glycosides in all four *P. nudicaule* cultivars, while strongly colored pelargonidin glycosides are restricted to apical petal areas of the orange and red cultivars and nudicaulins to apical petal areas of the yellow and orange flowers. This distribution pattern suggests that each group of compounds serves a different function. While the role in pigmentation is obvious, it may also be that some of the flavonoids have specific functions in growth and reproduction processes. Certain compounds may represent important metabolites that are present during petal development rather than biosynthesis products assigned to functions in the flowering stage. For example, it was already shown in yellow *P. nudicaule* flowers that pelargonidin glycosides decrease in favor of nudicaulin accumulation during petal development [9]. Future efforts should test these hypotheses and identify all substitution patterns of the flavonoids and carotenoids.

## 4. Materials and Methods

### 4.1. Plant Material

Seeds of *Papaver nudicaule* cultivars were purchased from Jelitto Staudensamen GmbH (“Summer Breeze”, yellow and orange) and Syringia (“Wonderland White” and “Matador Red”). The plants were reared in soil in the greenhouse facility of the Max Planck Institute for Chemical Ecology at temperatures between 21 °C and 23 °C during the day and between 19 °C and 21 °C during the night, with an average humidity of 55%. Phillips Sun-T Agro 400 Na lights were used to ensure a daily light period of 14 h. Plants were watered daily for 10 min. After sowing, a period of six to eight weeks passed before multiple buds developed on individual plants.

### 4.2. HPLC-PDA Analysis of Flavonoids and Nudicaulins

After freshly opened flowers were harvested, petals were separated, washed with deionized water and wiped dry, and the apical and basal areas were dissected. Every flower compartment was weighed and coarsely ground under liquid nitrogen. For extraction (Method 1), an amount of solvent in a 1:1 (*v*:*v*) ratio of water to methanol was added to achieve a uniform concentration of 1 mg of material per 10 µL of solvent (or, for capsules, per 5 µL). After 30 min of extraction in the ultrasonic bath, samples were centrifuged (4 °C, 13,200 rpm), and the supernatant was used for HPLC-PDA analysis. The entire procedure was carried out with three biological replicates.

The analytical HPLC-PDA system consisted of an Agilent series HP1100 (binary pump G1312A, auto sampler G1313A, and photodiode array detector G1315B, 200–700 nm) equipped with an EC250/4 Nucleodur C18 HTec column from Macherey-Nagel (5 µm; injection volume 20 µL). The method included a 21-min gradient from 20% to 67% of methanol in acidified water (0.1% trifluoroacetic acid) with a subsequent washing step (100% methanol) and equilibration to starting conditions. The flow rate was 1 mL·min^−1^, and the detection wavelengths were 211, 254, 281, 351, and 460 nm. The aglycone standards of kaempferol and pelargonidin chloride were purchased from Sigma-Aldrich.

### 4.3. UV/Vis Analysis of Carotenoids

For recording UV/Vis absorption spectra of carotenoids, the stamen and the upper capsule were separated, hexane was added, and the samples were homogenized using Bertin Minilys (60 s, full speed) (Method 2). After extraction in the ultrasonic bath for 3 min and centrifugation (10 °C, 13,200 rpm), spectra of the supernatant were measured against a hexane reference. Samples were applied inside a quartz cuvette with an internal width of 1 cm to a Jasco V-550 UV/Vis spectrophotometer.

## Figures and Tables

**Table 1:** Pigment distribution in the four studied flower parts.

*P. nudicaule* Cultivar	Apical Area	Basal Area	Capsule	Stamen
**White**	●	●	○ ●	● ● ●
**Yellow**	● ●	●	○ ●	● ● ●
**Orange**	● ● ●	●	○ ●	● ● ●
**Red**	● ●	●	○ ●	● ● ●

Colored dots indicate main compounds detected and colored circles (○) represent traces. ● Kaempferol glycosides, ● gossypetin glycosides, ● nudicaulins, ● pelargonidin glycosides, ● carotenoids.

## Supplementary Materials

The following are available online at www.mdpi.com/2223-7747/5/2/28/s1, Figure S1: Structures, mass spectra, and UV/Vis absorption spectra of representative glycosides of (**A**) kaempferol, (**B**) gossypetin, (**C**) nudicaulin, and (**D**) pelargonidin occurring in petals of *P. nudicaule*.

## References

[1] Parihar A., Grotewold E., Doseff A.I., Chen C. (2015). Flavonoid Dietetics: Mechanisms and Emerging Roles of Plant Nutraceuticals. Pigments in Fruits and Vegetables.

[2] Winkel-Shirley B. (2002). Biosynthesis of flavonoids and effects of stress. Curr. Opin. Plant Biol..

[3] Taylor L.P., Grotewold E. (2005). Flavonoids as developmental regulators. Curr. Opin. Plant Biol..

[4] Hanelt P. (1970). Die Typisierung von *Papaver nudicaule* L. und die Einordnung von *P. nudicaule* hort. non L.. Die Kulturpflanze.

[5] Robinson G.M., Robinson R. (1931). A survey of anthocyanins. I. Biochem. J..

[6] Robinson G.M., Robinson R. (1932). A survey of anthocyanins. II. Biochem. J..

[7] Cornuz G., Wyler H., Lauterwein J. (1981). Pelargonidin 3-malonylsophoroside from the red Iceland poppy, *Papaver nudicaule*. Phytochemistry.

[8] Tatsis E.C., Böhm H., Schneider B. (2013). Occurrence of nudicaulin structural variants in flowers of papaveraceous species. Phytochemistry.

[9] Warskulat A.-C., Tatsis E.C., Dudek B., Kai M., Lorenz S., Schneider B. (2016). Unprecedented utilization of pelargonidin and indole for the biosynthesis of plant indole alkaloids. ChemBioChem.

[10] Schliemann W., Schneider B., Wray V., Schmidt J., Nimtz M., Porzel A., Böhm H. (2006). Flavonols and an indole alkaloid skeleton bearing identical acylated glycosidic groups from yellow petals of *Papaver nudicaule*. Phytochemistry.

[11] Mol J., Grotewold E., Koes R. (1998). How genes paint flowers and seeds. Trends Plant Sci..

[12] Mo Y., Nagel C., Taylor L.P. (1992). Biochemical complementation of chalcone synthase mutants defines a role for flavonols in functional pollen. Proc. Natl. Acad. Sci. USA.

[13] Acheson R.M., Jenkins C.L., Harper J.L., McNaughton I.H. (1962). Floral pigments in *Papaver* and their significance in the systematics of the genus. New Phytol..

[14] Tétényi P., Rauter A.P., Palma F.B., Justino J., Araújo M.E., Santos S.P. (2002). Chemodifferentiation of Papavereae from coasts of the Black-Sea to the Atlantic. Natural Products in the New Millennium: Prospects and Industrial Application.

[15] Tatsis E.C., Schaumlöffel A., Warskulat A.C., Massiot G., Schneider B., Bringmann G. (2013). Nudicaulins, yellow flower pigments of *Papaver nudicaule*: Revised constitution and assignment of absolute configuration. Org. Lett..

[16] Suzuki H., Sasaki R., Ogata Y., Nakamura Y., Sakurai N., Kitajima M., Takayama H., Kanaya S., Aoki K., Shibata D. (2008). Metabolic profiling of flavonoids in *Lotus japonicus* using liquid chromatography Fourier transform ion cyclotron resonance mass spectrometry. Phytochemistry.

[17] Rodriguez-Amaya D.B., Kimura M. (2004). HarvestPlus Handbook for Carotenoid Analysi.

[18] Di Ferdinando M., Brunetti C., Fini A., Tattini M., Ahmad P., Prasad M. (2012). Flavonoids as antioxidants in plants under abiotic stresses. Abiotic Stress Responses in Plants.

[19] Cooper-Driver G.A. (2001). Contributions of Jeffrey Harborne and co-workers to the study of anthocyanins. Phytochemistry.

[20] Stich K., Halbwirth H., Wurst F., Forkmann G. (1997). UDP-glucose: Flavonol 7-*O*-glucosyltransferase activity in flower extracts of *Chrysanthemum segetum*. Z. Naturforsch. C.

[21] Wind O., Christensen S.B., Mølgaard P. (1998). Colouring agents in yellow and white flowered *Papaver radicatum* in Northern Greenland. Biochem. Syst. Ecol..

[22] Thompson W.R., Meinwald J., Aneshansley D., Eisner T. (1972). Flavonols: Pigments responsible for ultraviolet absorption in nectar guide of flower. Science.

[23] Pollak P.E., Vogt T., Mo Y., Taylor L.P. (1993). Chalcone synthase and flavonol accumulation in stigmas and anthers of *Petunia hybrida*. Plant. Physiol..

[24] Barrell P.J., Wakelin A.M., Gatehouse M.L., Lister C.E., Conner A.J. (2010). Inheritance and epistasis of loci influencing carotenoid content in petal and pollen color variants of California poppy (*Eschscholzia californica* Cham.). J. Hered..

