# Supplementary material for: The Occurrence of Flavonoids and Related Compounds in Flower Sections of Papaver nudicaule
Source: Plants (Basel). 2016 Jun 22;5(2):28. doi: 10.3390/plants5020028 (PMC4931408; doi:10.3390/plants5020028)
Supplement: Supplementary file 1 [file plants-05-00028-s001.pdf]

# Supplementary Materials: The Occurrence of Flavonoids and Related Compounds in Flower Sections of *Papaver nudicaule*

**Bettina Dudek, Anne-Christin Warskulat and Bernd Schneider***

## Mass Spectrometry

An Esquire 3000 ion trap mass spectrometer (Bruker Daltonics, Bremen, Germany) was used to measure the mass spectra in the positive mode in the range *m/z* 50–1500 with skimmer voltage ±33.9 V. Capillary exit voltage was ±100.6 V, capillary voltage 2500 V, nebulizer pressure 35 psi, drying gas 12.0 L $min^{-1}$, and gas temperature 350 °C.

## UV/Vis Spectroscopy

A photodiode array (PDA) detector (J&M Analytik AG, Aalen, Germany) was used for acquisition of UV/Vis absorption spectra (please note that the sensitivity of the detector was relatively low above 400 nm).

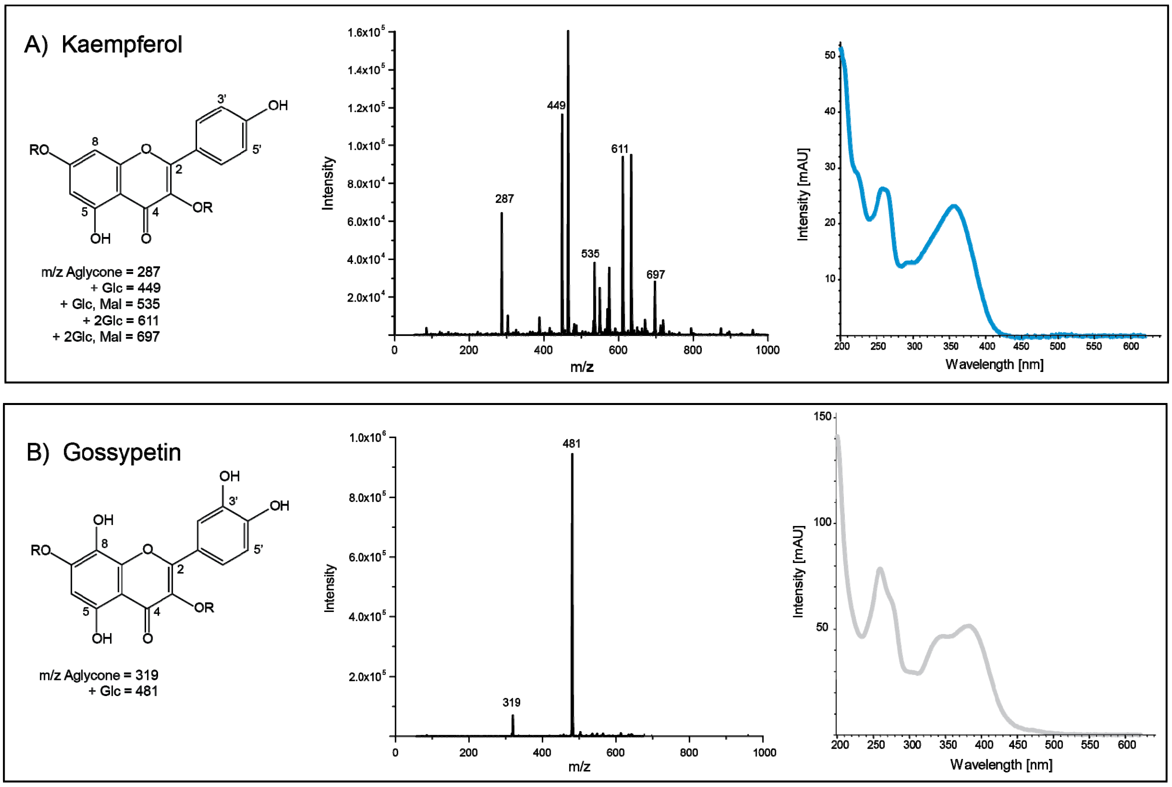


**Figure S1.** *Cont.*

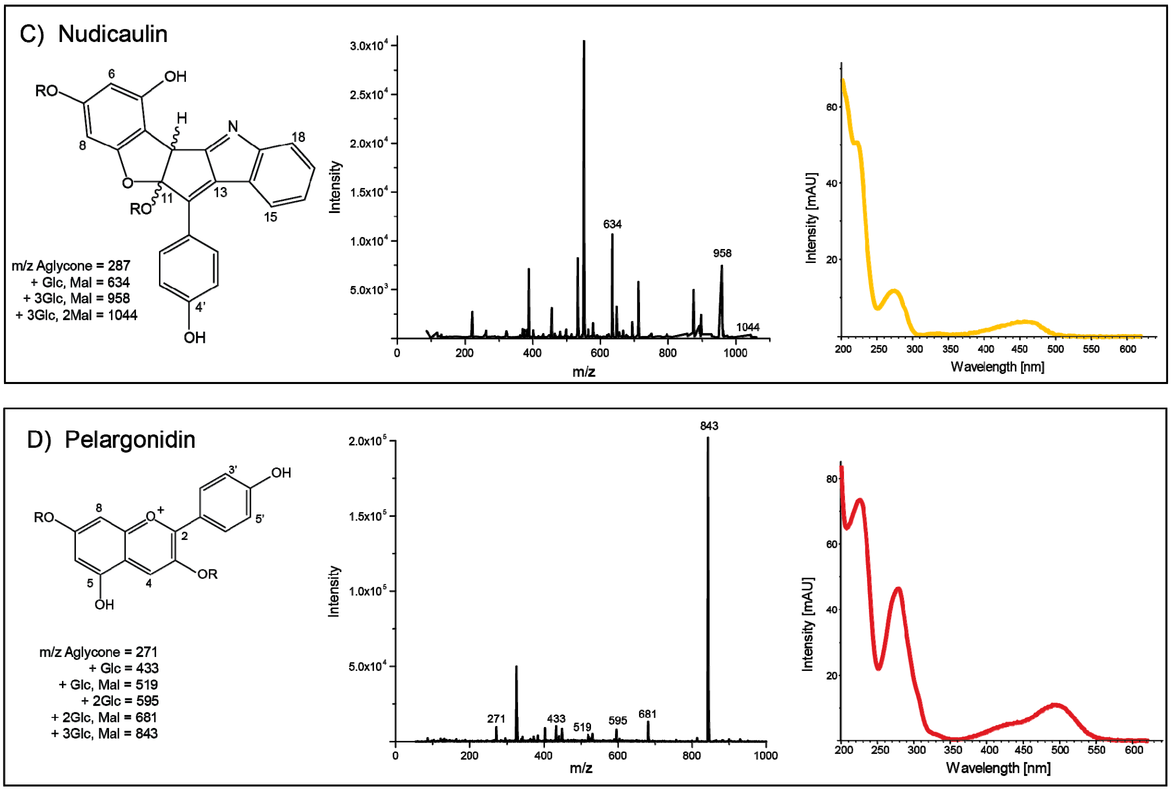


**Figure S1.** Structures, mass spectra and UV/Vis absorption spectra of representative glycosides of (**A**) kaempferol, (**B**) gossypetin, (**C**) nudicaulin, and (**D**) pelargonidin occurring in petals of *P. nudicaule.* Data are in agreement with previous studies [1–3]. *m/z* $[M+H]^+$; R indicate substitution by Glc, $Glc_2$, Glc-Mal or $Glc_2$-Mal; Glc = glycosyl; Mal = malonyl.

## References


1. Cornuz, G.; Wyler, H.; Lauterwein, J. Pelargonidin 3-malonylsophoroside from the red Iceland poppy, *Papaver nudicaule*. *Phytochemistry* **1981**, *20*, 1461–1462, doi:10.1016/0031-9422(81)80075-1.
2. Schliemann, W.; Schneider, B.; Wray, V.; Schmidt, J.; Nimtz, M.; Porzel, A.; Böhm, H. Flavonols and an indole alkaloid skeleton bearing identical acylated glycosidic groups from yellow petals of *Papaver nudicaule*. *Phytochemistry* **2006**, *67*, 191–201, doi:10.1016/j.phytochem.2005.11.002.
3. Tatsis, E.C.; Böhm, H.; Schneider, B. Occurrence of nudicaulin structural variants in flowers of papaveraceous species. *Phytochemistry* **2013**, *92*, 105–112, doi:10.1016/j.phytochem.2013.04.011.
